# Roles of Iroquois Transcription Factors in Kidney Development

**DOI:** 10.4172/2168-9296.1000131

**Published:** 2014-01-01

**Authors:** Amanda N. Marra, Rebecca A. Wingert

**Affiliations:** Department of Biological Sciences, University of Notre Dame, Notre Dame, USA

**Keywords:** Kidney, Nephron, Nephrogenesis, Segmentation, CAKUT, Pronephros, Mesonephros, Metanephros, Frog, Zebrafish, Mammal

## Abstract

Congenital anomalies of the kidney and urinary tract (CAKUT) affect 1/500 live births. CAKUT lead to end stage renal failure in children, and are associated with high morbidity rates. Understanding the mechanisms of kidney development, and that of other associated urogenital tissues, is crucial to the prevention and treatment of CAKUT. The kidney arises from self-renewing mesenchymal renal stem cells that produce nephrons, which are the principal functional units of the organ. To date, the genetic and cellular mechanisms that control nephrogenesis have remained poorly understood. In recent years, developmental studies using amphibians and zebrafish have revealed that their simple embryonic kidney, known as the pronephros, is a useful paradigm for comparative studies of nephron ontogeny. Here, we discuss the new found roles for *Iroquois* transcription factors in pronephric nephron patterning, and explore the relevance of these findings for kidney development in humans.

## Introduction to Kidney Ontogeny and Composition

Kidney development in vertebrates is an elaborate process that involves the formation and progressive degradation of up to three kidney forms known as the pronephros, mesonephros, and metanephros [1]. Each kidney form is architecturally more complex than its predecessor, but each form nevertheless consists of fundamentally similar structural and functional units known as nephrons [1]. The nephrons perform essential regulatory roles in the body, collecting metabolic waste for urinary excretion and working to maintain water homeostasis [2].

The pronephros, mesonephros, and metanephros kidney forms carry out renal functions to varying degrees during the different stages of development and adult life of vertebrate species. In higher vertebrates like birds, reptiles, and mammals, the pronephros is a vestigial organ with little to no function. In these animals, the mesonephros functions transiently during the gestation stages as the metanephros takes shape, then degrades when the developed metanephros becomes functional. In lower vertebrates like fish and amphibians, however, the pronephros is fully functional during embryonic and/or juvenile stages, and the mesonephros is the final kidney to form and serves as the adult organ. Across species, the number and arrangement of nephrons present in each kidney form differs widely. Pronephros kidney forms tend to be anatomically simple, and contain the fewest number and simplest arrangements of nephrons. In contrast, mesonephros and metanephros kidney structures contain increased nephron endowments and more complicated branching or otherwise arborized arrangements of the nephrons around a drainage system.

Nephrons in all of these vertebrate kidney structures are comprised of three conserved parts: a blood filter, tubule, and duct [2]. Each nephron part is made up of several highly specialized epithelial cell types [2]. The blood filter prevents circulatory cells (e.g. erythrocytes and leukocytes) and large proteins from entering the nephron tubule, but does not discriminate among low molecular weight metabolites that will pass into the tubule [2]. The tubule is organized into a series of discrete proximal, intermediate and distal segments that carry out unique jobs, and in turn each of these segment regions consists of differentiated cells with unique ultrastructural and molecular features when compared to neighboring regions [2]. The functions of the tubule encompass the reabsorption and/or secretion of glucose, amino acids, and various salts, which ultimately preserves essential nutrients from excretion and maintains a stable internal environment [2]. Finally, the duct transports the urine out of the kidney, and the cells that comprise the duct perform fine-tuning of electrolytes through the reabsorption of salt and water [2]. Various cell types in the nephron and cells located in the interstitial space between nephrons additionally work to produce and secrete hormones that control blood pressure and hematopoiesis, such as renin and erythropoietin [3].

The integrity of kidney nephrons is absolutely essential for normal renal function in vertebrates, and the abrogation of nephron functionality leads to various forms of kidney disease [4]. How kidney cells are made during development is hypothesized to hold the key for how replacement renal cells might be generated through regenerative medicine to treat kidney disease [5,6]. Currently, there is a growing incidence of acute and chronic kidney diseases [3,4]. There is also great medical significance in understanding kidney formation, as kidney development defects are relatively common. Congenital abnormalities of the kidney and urinary tract (CAKUT) affect 1/500 children when they are born, and represent 20–30% of prenatal anomalies [7,8]. The defects within the clinical spectrum of CAKUT are incredibly diverse, and include (i) *agenesis*, or failure of the kidneys to form, (ii) *hypoplasia*, the formation of kidneys that are reduced in size and/or overall nephron number, and (iii) *dysplasia*, in which the kidney is comprised of abnormal structures such as cystic nephrons [7–10]. Identifying the key genes that control different aspects of kidney development can be used to determine the origins of CAKUT conditions.

## Animal Models of Nephron Development

There have been tremendous strides in understanding early events in renal development through the genetic analysis of mammalian kidney development using the mouse model system, as briefly discussed in the following section [11]. However, the mechanisms of nephron development, particularly how segmental domains emerge during nephrogenesis, have remained poorly understood with a small number of signals and molecular targets identified to date [8,12–14]. The striking commonalities of nephron composition and excretory tasks that exist amongst species have become increasingly appreciated in recent years [15,16]. These observations have spurred a burgeoning interest and expansion in basic research efforts to study the developmental biology of renal stem cells by utilizing diverse vertebrate models [15,16]. In fact, several invertebrates models have been utilized as well, including the *Drosophila* fruit fly and the nematode *C. elegans*, which further highlights the amazing degree of conservation among excretory cell types across the phylogenetic tree [15,16].

**Figure 1:**
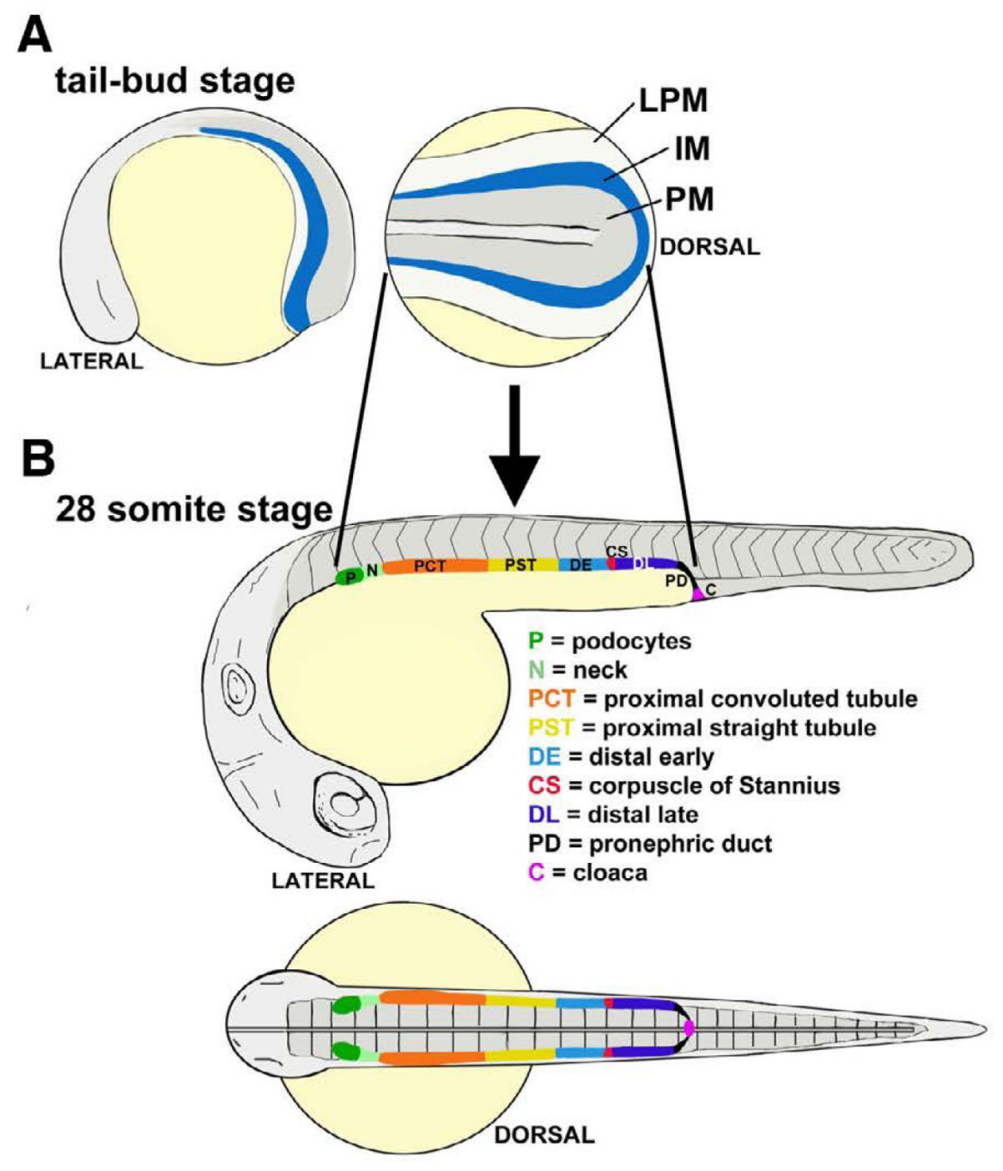
Pronephros development in the zebrafish (A) The renal progenitor field emerges from the intermediate mesoderm (IM) which arises between the paraxial mesoderm (PM) and the lateral plate mesoderm (LPM). (B) The renal progenitor fields give rise to a pair of bilateral nephrons that are comprised of a series of discrete cell types. (Images adapted from [18] with author rights).

Of relevance for this review, the pronephros in both the frog *Xenopus laevis* and the zebrafish *Danio rerio* have emerged as useful systems to delineate the mechanisms of nephron segmentation, largely based on new-found appreciation for the cellular and molecular similarities shared between nephrons in these species and mammals [17,18]. Further, the relatively simple anatomy of these pronephros kidneys facilitates nephrogenesis analysis (Figure 1). Several recent studies have identified roles for the *Iroquois (Irx)* transcription factor *Irx3* in the proximodistal segmentation of the vertebrate nephron using the frog [19] and zebrafish pronephros [20]. Here, we provide a brief overview of renal lineage development to set the stage for a detailed discussion of *Irx* gene form and function in nephron pattern formation. We discuss the series of findings that have established crucial roles for *Irx3* in pronephros segment patterning based on several studies in frog and zebrafish embryos. Finally, we discuss the relevance of these findings to mammalian metanephros nephrogenesis.

## Setting the Stage: An Overview of Renal Lineage Specification and Patterning in Vertebrates

As previously discussed, kidneys across species utilize nephrons as their basic structural and functional building block regardless of the architectural variations between kidney types. During ontogeny, renal lineages derive from the intermediate mesoderm (IM) in the embryo. To date, the early events in kidney specification have been extensively characterized through genetic studies in the murine metanephric kidney [3,11]. In mammals, IM cell type specification involves the activity of several transcription factors, including *Osr1, Eya1, Pax2*, though the precise relationships between these and other genes is still an active area of investigation [11]. IM specification leads to emergence of two major lineages: the so-called nephric duct and a population of Metanephric Mesenchyme (MM) cells that will undergo complex interactions to form the metanephros [3,11]. Formation of the nephric duct involves many genes, among them *Pax2/8* and *Lhx1* [11]. Essential transcription factors that support MM development include *Osr1, Pax2, Wt1* and several *Hox* genes [11]. The MM becomes delineated into sub-compartments that include vascular progenitors, a stromal progenitor population, and a population of renal stem cells [11]. Stromal progenitors are identified based on their expression of *Foxd1*, and give rise to cell types located in the interstitial space between nephrons [11]. Renal stem cells that express *Six2* and *Cited1* undergo proliferation and self-renewal, and their offspring produce nephrons through an intricate process termed nephrogenesis.

Nephrogenesis is initiated when a cluster of renal stem cell offspring, or renal progenitors, aggregates and undergoes a mesenchymal to epithelial transition, forming a renal vesicle (RV) [3,11]. The RV is a hollow structure that elongates through further cell proliferation and undergoes morphogenesis to become the nephron tubule [3]. The RV will lengthen into a comma-shaped body, followed by an S-shaped body before finally transitioning into a complete nephron [3]. Nephrogenesis also involves the specification and pattern formation of renal progenitors into the many different cell types found in each nephron [12–14]. This segmentation of the nephron and the subsequent proper differentiation of the many specialized epithelial is crucial for normal renal development. While several transcription factor genes and signaling pathways have been identified as essential components in nephron cell lineage development, the cast of players is considered incomplete and the mechanisms of nephron patterning remain poorly understood [12–14]. Several recent studies have implicated important nephrogenesis roles for a member of the *Irx* gene family, *Irx3*, which are discussed in the subsequent sections of this review.

## Overview of the *Irx* Gene Family

The Iroquois (known both as *Iro/Irx*) genes are a conserved family of homeo-domain transcription factors that are well known for their roles in the tissue patterning and cell type specification [21,22]. The *Irx* genes were discovered in *Drosophila* in a screen to identify factors responsible for the patterning of sensory structures, such as the bristles located on the thorax of the fly [23,24], and include a cluster of three genes known as *araucan*, *caupolican*, and *mirror* [21]. Since this time, they have been assigned a broad number of roles in the organization of cell types in both invertebrates and vertebrates. For example, studies have demonstrated that *Irx* genes are required to pattern tissues such as the eye primordium of *Drosophila* [25], the vertebrate brain [26], spinal cord [27], and heart [28]. *Irx* factors can act as transcriptional activators and repressors [21,22]. In vertebrates such as *Xenopus* and the mouse there are six members of the *Irx* family divided in two clusters: cluster A (*Irx1, Irx2* and *Irx4*) and cluster B (*Irx3, Irx5* and *Irx6*) [29,30]. Teleost fish, likely due to a genome duplication event that occurred after the divergence of teleosts from tetrapods, possess more *Irx* genes. Thus far, a total of 10 *Irx* genes were reported in puffer fish (*Fugu rubripes, Tetraodon nigroviridis*), and 11 have been detected in the zebrafish [31].

### Discovery that *Irx3* is Essential for Pronephros Segmentation in the Frog

The frog has been used historically as a powerful model to delineate the embryological mechanisms involved in organ induction [32,33]. The discovery that the frog pronephros contained proximodistal segmentation that likened it to the segmented mammalian nephron led to the further application of the *Xenopus* model to interrogate segmentation mechanisms [34]. Several reports noted the expression of *Irx* genes in the frog kidney [35] and mouse kidney [36], but the function of this family of genes in the kidney went unexplored until a group led by Reggiani, *et al.* hypothesized that *Irx* genes may direct nephron patterning [19].

To explore their hypothesis, Reggiani, *et al.*, first surveyed *Irx* gene expression during *Xenopus* pronephros development [19]. The *Xenopus* pronephric nephron contains four major tubule segments known as the proximal early tubule (PE), proximal late tubule (PL), distal early (DE) and distal late tubule (DT), which is followed by a duct [34]. Based on the analysis of gene expression patterns, the segments have also been proposed to be more elaborate—i.e. to be further sub-compartmentalized [19,37]. In the more elaborate schema, the proximal tubule is further organized into three segments, rather than two: PT1, PT2 and PT3 [19,37]. In addition, an intermediate tubule with two different regions, the IT1 followed by IT2, is proposed to sit between the proximal and distal segments [19,37]. These segments can be highlighted using combinations of mammalian kidney gene orthologs, such as the *solute carrier (Slc)* family of genes [19,37].

Gene expression analyses revealed that several *Irx* genes were regionally expressed in renal progenitors during nephrogenesis [19]. During pronephric nephron development in *Xenopus*, *Irx* expression is highly specified and confined to the central region. Notably, three specific *Irx* factors are present during frog pronephros development: *Irx1, Irx2*, and *Irx3* [19]. Interestingly, *Irx3* expression is specific to the PT3, IT1 and IT2 nephron segments as early as stage 25 of the developing embryo [19]. The expression of *Irx3* appears before both *Irx1* and *Irx2*, which are subsequently expressed solely in the IT1 [19]. The expression of *Irx3* in *Xenopus* begins to decline at stage 35/36 [19]. However, *Irx1* and *Irx2* expression remains strong [19].

To determine the role(s) of these *Irx* genes, Reggiani, *et al.* performed a series of loss of function studies using antisense morpholinos (MO) [19]. For each *Irx* gene in question, two distinct MOs were used [19]. All six Irx-MOs were proven to inhibit *Irx* mRNA translation in a concentration-dependent fashion [19]. Also, each Irx-MO was specific to its *Irx* gene target [19]. For example, the two Irx3-MOs did not affect *Irx1* or *Irx2* translation [19].

Next, Reggiani *et al.* evaluated nephron segment pattern in the pronephros through knockdown experiments utilizing *Irx*-targeting MOs into single V2 blastomeres of 8-cell stage embryos [19]. At this stage, it has been documented that MOs can target the prospective pronephric anlage [38]. After injection, the embryos were examined for physical abnormalities as well as for marker gene expression within the pronephros region via *in situ* hybridization. Simultaneous knockdown of *Irx1*, *Irx2* and *Irx3* resulted in embryos with clear developmental defects [19]. One significant defect was the unilateral shortening of the body axes, which precluded the analysis of possible pronephric phenotypes [19]. Thus, the simultaneous loss of *Irx1*, *Irx2* and *Irx3* promoted abnormal development of the embryo as a whole. However, loss of *Irx1* or *Irx2* alone, or the combination of *Irx1* and *Irx2* together, had no physical affect on development, or on the expression of pronephric marker genes [19]. Conversely, injection of an Irx3-MO resulted in developmental defects specific to the pronephric kidney of the majority of the embryos assayed [19]. Further, a second independent morpholino, Irx3 (2)-MO, produced similar phenotypes [19]. Interestingly, a small fraction of both the Irx3-MO and Irx3 (2)-MO injected embryos had complete pronephric kidney loss accompanied by other developmental malformations [19].

In the Irx3-Mo injected embryos that appeared to have a normal phenotype, *in situ* analysis was done to probe for evidence of defects in nephron organization [19]. In these *Irx3* knockdowns, *Pax2* expression revealed the presence of pronephric kidneys, indicating that the specification of the pronephric fate had occurred during gastrulation [19]. However, the majority of the embryos possessed defects in the intermediate tubule, characterized by abnormal morphology of the looped central domain [19]. Staining for segment markers showed the loss of IT1 and IT2, as well as decreased size of the PT3 [19]. Embryos injected with Irx3 (mp)-MO, a control, demonstrated no expression loss of any segment markers [19]. Interestingly, *Irx3* knockdown also resulted in a loss of both *Irx1* and *Irx2* expression in the PT3, IT1 and IT2 segments [19]. However, *Irx3* knockdown did not alter pronephric terminal differentiation, nor did it affect the expression of *Irx3* in the same embryos when allowed to age [19].

To determine if *Irx3* specifies intermediate tubule fate, Reggiani *et al.* performed gain of function experiments [19]. To do this, *Irx3* mRNA was injected into either one blastomere of two-cell-stage embryos or the V2 blastomere of eight-cell-stage embryos [19]. The embryos were raised to the stage 39 and evaluated for the presence of the IT and DE marker *Slc12a1* via *in situ* hybridization [19]. About half of the injected embryos demonstrated developmental defects as serious as abnormal neural tube closure [19]. In about 10% of the embryos that showed no phenotypical defects, the formation of ectopic *Slc12a1*-expressing tubule tissues was seen [19]. These ectopic tissues were solely located in the intermediate mesoderm posterior to the pronephric kidney, suggesting that *Irx3* over expression is only able to promote the change of intermediate mesoderm to IT/DE tubule tissue [19].

Taken together, these studies established that *Irx3* function is essential for segmentation in the amphibian pronephros, and that *Irx3* is required to induce the expression of *Irx1* and *Irx2* [19]. Together, all three of these genes are important for patterning of the pronephric kidney. Several intriguing questions about the frog pronephros have not yet been resolved. The expression of *Irx3* is clearly confined to the central region of the developing pronephric nephron, but it is difficult to explicitly define *Irx3* expression to the intermediate tubule from this study [19]. The *Slc* markers used to outline the intermediate and DE pronephric segments largely overlapped. Thus while it is convincing that *Irx3* marking the tubule regions downstream of the proximal segments, the existence of the intermediate tubule would be strengthened if specific intermediate tubule markers could be identified. Notably, the same research group reported a large panel of 112 pronephros markers subsequent to the *Irx* study [37]. This collection contains a number of intriguing markers that show expression in unique combinations of nephron regions, including *Cldn8* in the proposed IT2 segment [37]. However, only one IT1 marker, *Kcnj1*, was reported in this study, and this transcript is also expressed throughout the distal tubule and collecting duct [37]. Additional IT markers have not been reported to date. Nevertheless it would be intriguing to assay whether *Irx3* gain of function also triggers the ectopic expression of *Cldn8* and/or *Kcnj1*, as evidence of such a finding could be used to further identify the cell type(s) that *Irx3* promotes.

In a subsequent 2008 study from Alarcon, *et al., Irx1* and *Irx3* were found to be requisite to maintain the identity of the territory that gives rise to the pronephros [38]. Gain or loss of Irx function during neurula stages led to the expansion or reduction of the pronephros field [38]. These findings suggest that *Irx* gene activity in amphibians is crucial to maintain the size of the IM field that produces the embryonic kidney-thus highlighting an early role for *Irx* activity prior to the events of nephron segmentation. If and how this finding relates to mammalian renal lineage specification has not yet been the subject of further investigation.

## *Irx3* Function in Distal Tubule Segmentation in the Zebrafish Pronephros

Studies in zebrafish have suggested that one *irx3* paralog, *irx3b*, plays a role in segmentation through regulating segment boundaries and ensuring distal segment differentiation [20]. Over the last several decades, the zebrafish has steadily emerged as a powerful model organism for developmental genetics [39,40]. A landmark study first described the zebrafish embryo pronephros, demonstrating that it was comprised of two nephrons similar to the *Xenopus* embryo, but instead possessed an integrated blood filter that was more structurally reminiscent of the mammalian blood filter [41]. The nephrons in the zebrafish nephron were subsequently discovered to possess a segmental composition [17,18], with at least eight different cell types that included a series of proximal and distal segments (Figure 1). Among the tubule segments, there are several proximal and distal regions: a proximal convoluted tubule (PCT), proximal straight tubule (PST), followed by a distal early (DE) and distal late (DL) segment [42], named based on their similarity to the *Xenopus* pronephros organization [34].

Since this discovery, the zebrafish pronephros has been increasingly used to pursue developmental studies on renal progenitor patterning and nephrogenesis [17,18,20,43,44], as well as to model acute kidney injury and regeneration [45–47]. In terms of nephron formation, the pronephros is also a valuable model for two major reasons. First, the nephrons are linear at early developmental stages, and this enables high-resolution analysis of segmental domains in comparison to other axial landmarks (Figure 1) [20,42]. Simply put, the architecture and anatomical arrangement of the pronephric nephrons are highly amenable to evaluating the size and domain of each segment. Second, the zebrafish model permits forward and reverse genetics, thus enabling powerful modes of gene discovery and analysis.

**Figure 2:**
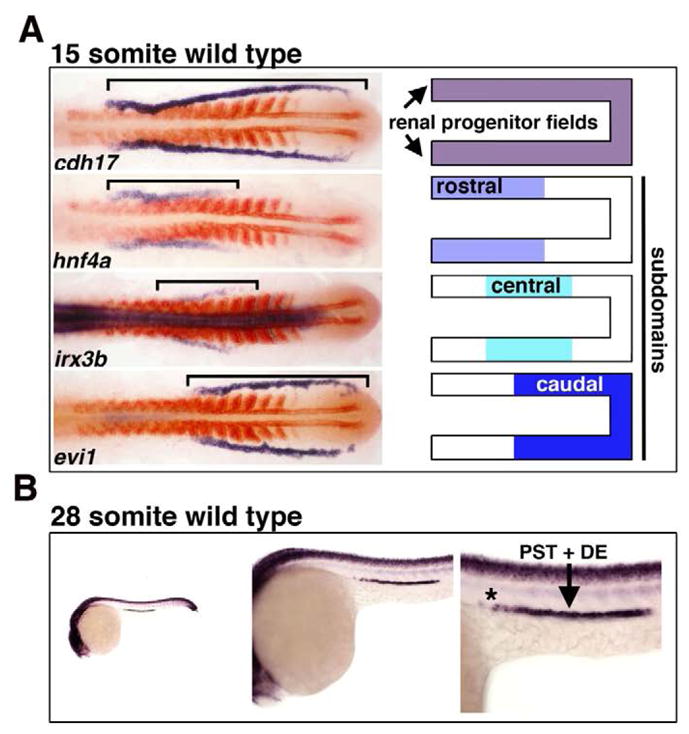
*irx3b* expression in the zebrafish pronephros Whole mount *in situ* hybridization was performed to label *irx3b* (purple) or other kidney markers (*cdh17*, *hnf4a*, and *evi1 (*now known as *mecom,* [51])) (purple), along with *myoD1* (red) used to label the somites. (A) *irx3b* is regionally expressed in the central domain of the renal progenitor field at the 15 somite stage of development. (Images of select 15 somite stage embryos adapted with author rights from [20]). (B) At the 28 somite stage, *irx3b* transcripts demarcate the central domain of the segmented nephron, and are most strongly expressed in the PST and DE segment domains.

In a recent study, the role of *irx3b* was assessed in the zebrafish pronephros through gene expression and knockdown studies [20]. In the developing zebrafish, the renal progenitors can be detected just after gastrulation in bilateral stripes of IM located on either side of the embryonic midline (Figure 1A) [20]. As somitogenesis occurs, the renal progenitor field displays regionalized patterns of gene expression that suggest the emergence of rostral and caudal domains [20]. Several hours later, at the 15 somite stage of development, a central domain emerges that is demarcated by *irx3b* (Figure 2A) [20]. *irx3b* transcripts continue to be expressed by centrally-located renal progenitors through to the 28 somite stage (Figure 2B). At this stage, the discrete nephron segment domains are formed, and can be visualized based on the restricted expression of specific solute transporters [20]. *irx3b* transcripts persist at high levels in the PST and DE segments throughout this time (which are the last proximal segment and the first distal segment) [20]. In addition, low levels of *irx3b* transcripts are detectable in the locale of the PCT that neighbors the PST (note the asterisk in Figure 2B) [20].

**Figure 3:**
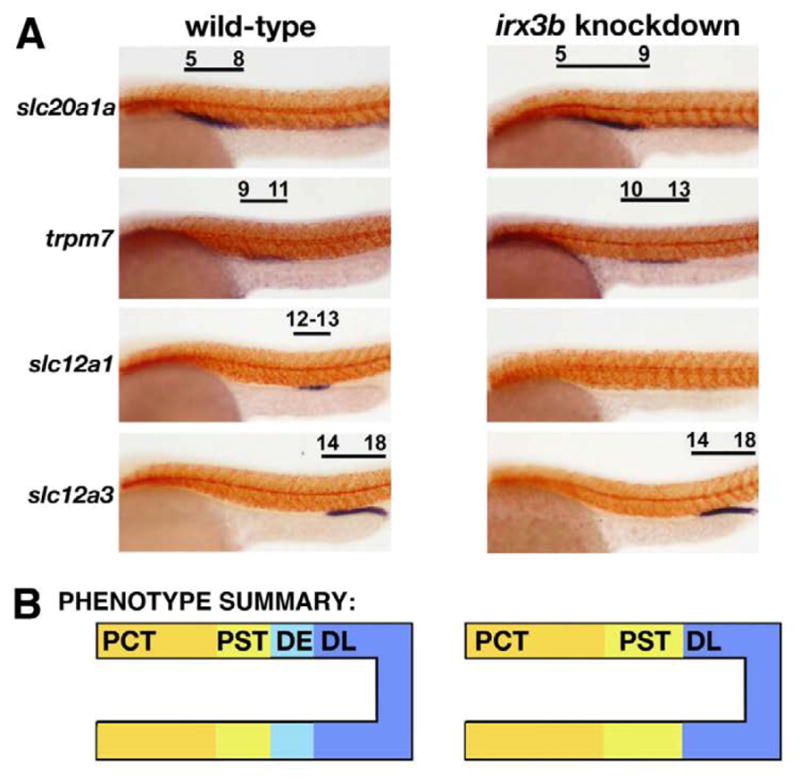
*irx3b* knockdown in the zebrafish embryo leads to abrogation of the distal segment known as the DE, and concomitant expansion of the proximal nephron segments (A) Whole mount *in situ* hybridization was performed to assess tubule segment composition in wild type embryos compared to *irx3b* knockdowns. In *irx3b* knockdowns, the expression domains of the PCT and PST markers *slc20a1a* and *trpm7* are each expanded by one somite length, while the DE is absent and the DL is unchanged. (Images of selected 15 somite stage embryos adapted with author rights from [20]). (B) Summary of the segmental composition in wild type and *irx3b* knockdowns.

Interestingly, the loss of *irx3b* function in zebrafish, which was assessed by injecting one-cell stage embryos with morpholinos to abrogate protein expression, led to the specific loss of the DE segment (Figure 3) [20]. This suggests that *irx3b* is essential for DE formation. In place of the DE, slightly expanded domains of both the PCT and PST developed (Figure 3) [20]. This could indicate that *irx3b* functions to modulate the boundary of the PCT/PST, a notion supported by the low level of *irx3b* transcripts that are detectable across the PCT/PST border region in wild type zebrafish embryos (Figure 2B). Further, knockdown of *irx3b* was associated with expansion of the Corpuscle of Stannius (CS), though this observation has yet to be further analyzed [20]. Taken together with the data from *Xenopus*, these findings suggest that a conserved role of *Irx3/irx3b* is to direct the proper development of the segmental identities located in central domains of the nephron. However, future studies are needed to test the function of other *irx* family members in the zebrafish. Further, it remains unknown if the loss of *irx3b* is associated with any changes in the expression of other *irx* genes in the zebrafish pronephros.

## Relevance of *Irx* Gene Activity during Mammalian Nephrogenesis

Gene expression studies of the mammalian kidney performed by Reggiani, *et al.* provide some basis for the hypothesis that *Irx* functions may be conserved between lower vertebrates and mammals [19]. During mouse metanephros ontogeny, several *Irx* genes are expressed during nephrogenesis when the nephron precursors are undergoing proliferation and elongation in so-called comma and S-shaped formations [19]. The authors found that *Irx3* is modestly expressed in the early comma shaped nephron [19]. In the S-shaped nephron, the central region of cells expressed *Irx1, Irx2*, and lower levels of *Irx3* [19]. In the adult kidney, Reggiani, *et al.* also detected regionalized *Irx* gene expression, with all three genes in the S3 segment of the proximal tubule, while *Irx1* and *Irx2* transcripts were found in the first section of the distal tubule known as the thick ascending limb [19]. These domains are certainly reminiscent of the regionalized location for the corresponding *Irx3/irx3b* orthologues in the central nephron segments in *Xenopus* and zebrafish, respectively. Although these expression patterns are suggestive, functional analysis of *Irx* genes is necessary in the mammalian kidney. Interestingly, recent studies of the *hepatocyte nuclear factor 1B (Hnf1b)* transcription factor in both zebrafish and mammals have implicated Irx genes acting downstream during nephrogenesis [48–50]. In zebrafish, loss of *hnf1b* gene activity by dual knockdown of the *hnf1ba/b* paralogs led to the loss of *irx3b* expression, and *irx3b* was reciprocally found to be required to maintain *hnf1ba/b* expression in the pronephros [48]. In the mouse, the inactivation of *hnf1b* is associated with drastic tubular defects, including the loss of intermediate and distal segments that correlated with reductions in the expression of *Irx1* and *Irx2* [49,50]. Further assessment of *Irx* gene expression in these models is needed. For example, performing the conditional inactivation of single *Irx* genes and combinations of *Irx* genes are some of the next crucial steps in elucidating their functional roles and determining whether they share redundant functions during kidney development.

## Conclusions and Future Perspectives

Nephrons have three fundamental parts, a blood filter, tubule comprised of numerous functional segments, and a duct. This fundamental structure of the nephron is broadly conserved in both in form and function across organisms that are evolutionarily quite diverse. At present, there is only a rudimentary knowledge of the gene regulatory networks that control nephrogenesis, particularly those involved in the pattern formation through which segment identities emerge. Uncovering these networks is vital to piece together the mechanisms of renal development. The pronephros in the frog and zebrafish shares fundamental similarities in nephron segment composition and organization with higher vertebrates. Several studies of the Irx gene family have now implicated these genes as players in orchestrating segment patterning. The findings of Reggiani, *et al.* suggest a model in which *Irx3* regulates the expression of *Irx1* and *Irx2*, and is necessary for the development of centrally-located segments in the frog nephron [19]. In zebrafish, the loss of *irx3b* is associated with a block in distal segment differentiation [20]. It is reasonable to hypothesize a functional role for the orthologues of these Irx genes during mammalian nephron development based on the appearance of transcripts encoding *Irx1, Irx2*, and *Irx3* in elongating metanephric kidney nephrons in the mouse, and emerging data from the study of *hnf1b* mouse knockout models [19,49,50].

Taken together, lessons learned from investigations of the pronephros have promise to help identify candidates that may be involved in nephron segmentation. In particular, the genetic tractability of the zebrafish model provides several unique opportunities for the identification and functional testing of nephrogenesis factors, as exemplified by recent studies [20,48,51]. The zebrafish model can be used to conduct forward and reverse genetic screens, as well as chemical genetic screens, to ascertain essential components of nephron pattern formation [39,45]. Both the zebrafish and frog embryos can be utilized for rapid functional assessments of renal genes through loss- and gain-of-function interrogations. Thus, continued use of these models has significant potential to provide new information about the mechanisms of nephron segmentation, and may someday lead to insights applicable toward CAKUT therapies.
